# Influence of age, gender and educational level on performance in the Brief Cognitive Battery-Edu

**DOI:** 10.1590/S1980-57642009DN20200007

**Published:** 2008

**Authors:** Ricardo Nitrini, Sonia Maria Dozzi Brucki, Jerusa Smid, Maria Teresa Carthery-Goulart, Anghinah Renato, Renata Areza-Fegyveres, Valeria Santoro Bahia, Antonio Eduardo Damin, Ana Paula Formigoni, Norberto Anízio Ferreira Frota, Carla Guariglia, Alessandro F. Jacinto, Eliane Mayme Kato, Edson Erasmo Pereira Lima, Daniel Moreira, Ana Beatriz Nóbrega, Claudia Sellitto Porto, Mirna Lie Hosogi Senaha, Mari Nilva M. Silva, Juliana Neri Souza-Talarico, Marcia Radanovic, Leticia Lessa Mansur

**Affiliations:** Behavioral and Cognitive Neurology Unit, Department of Neurology, University of São Paulo School of Medicine. Grupo de Neurologia Cognitiva e do Comportamento, Departamento de Neurologia, Faculdade de Medicina da Universidade de São Paulo.

**Keywords:** neuropsychological tests, education, aging, Brief Cognitive Battery

## Abstract

The Brief Cognitive Battery-Edu (BCB-Edu) contains nine tests, seven of which are related to the memory of drawings, and has good accuracy in the diagnosis of cognitive impairment.

**Objectives:**

To evaluate the influence of age, gender and educational level on the performance in tests related to memory of drawings of the BCB-Edu in healthy subjects.

**Methods:**

Participants were adult volunteers; exclusion criteria were illiteracy, neurologic or psychiatric disorders, visual or hearing impairment, untreated chronic clinical conditions, alcoholism, use of drugs, and for those aged 65 or over, an informant report of cognitive or functional impairment. We evaluated 325 individuals (207 women), with a mean age of 47.1 (±16.8) years, ranging from 19 to 81, and a mean of 9.8 (±5.0) schooling-years. Univariate analyses, correlations and logistic regression were employed (α=0.05).

**Results:**

There were significant negative correlations between age and the scores in four of the seven tests. However, schooling-years were positively correlated to the scores, where schooling-years decreased with age in this sample (rho= -0.323; p<0.001). Logistic regression confirmed that gender influenced the learning of drawings, where women performed better, while age influenced incidental memory, immediate memory, learning and delayed recall of the drawings, and schooling-years influenced visual identification, immediate memory, learning, delayed recall and recognition of the drawings.

**Conclusion:**

Gender, age and education influence the performance on the memory of drawings of the BCB-Edu, although the extent of these influences differs according to the nature of the test.

## Abstract (pt)

A Bateria Breve de Rastreio Cognitivo - Edu (BBRC-Edu) contém nove testes, sete dos quais relacionados à memória de desenhos. Tem uma boa acurácia no diagnóstico de comprometimento cognitivo.

**Objetivos:**

Avaliar a influência da idade, sexo e nível educacional no desempenho nos testes relacionados à memória de desenhos da BBRC-Edu em sujeitos saudáveis.

**Métodos:**

Os participantes foram voluntários; os critérios de exclusão foram: analfabetismo, doenças neurológicas ou psiquiátricas, perda auditiva ou visual, condições clínicas descompensadas, etilismo, uso de drogas, e para aqueles com idade igual ou superior a 65 anos, informação de acompanhante de presença de comprometimento cognitivo ou funcional. Foram avaliados 325 indivíduos (207 mulheres), com idade média de 47.1 (±16.8) anos, variando de 19 a 81 nos, média de 9.8 (±5.0) anos de escolaridade. Análise univariada, correlações e regressão logística foram empregadas (α=0.05).

**Resultados:**

Houve correlações negativas significativas entre idade e os escores de quatro dos sete testes. A escolaridade correlacionou-se positivamente aos escores, embora a escolaridade diminua com a idade nesta amostra (rho= -0.323; p<0.001). Regressão Logística confirmou que o gênero tem influência no aprendizado, com melhor desempenho entre as mulheres, enquanto a idade influenciou a memória incidental, memória imediata, aprendizado e evocação das figuras; e a escolaridade influenciou a identificação, memória imediata, aprendizado, evocação e reconhecimento.

**Conclusão:**

Gênero, idade e educação tiveram influência no desempenho na memória de figuras da BBRC-Edu, embora de modo diferente entre os tipos de teste.

The Brief Cognitive Battery-Edu (BCB-Edu) has good accuracy in the diagnosis of cognitive impairment in populations with a heterogeneous educational background.^1,2^ It was designed for evaluating the cognitive performance of low-educated individuals, including illiterates.^3,4^ BCB-Edu comprises nine tests, seven of which are related to the identification, naming, and memorization of drawings of 10 simple objects. A sheet of paper with 10 drawings is shown to the participant who is requested to name the objects. The encoding or acquisition is performed in three steps (incidental memory, immediate memory and learning) and is followed, after an interval with a heterogeneous interference task, by the delayed recall of the objects, which is carried out without cues, and through recognition of the 10 drawings among other 10 distractors. The other two tests, which are applied during the interference interval, are the verbal fluency (animals in one minute) and clock-drawing tests.^3,4^

It has been possible to verify that this simple and brief battery is not heavily influenced by educational level, particularly the delayed recall test of the 10 drawings.^2,4^ It is well known that delayed recall tests are the most sensitive test for the diagnosis of dementia in Alzheimer’s disease.^5^ In previous papers we have shown that the delayed recall of the BCB-Edu is more appropriate than the delayed recall of a list of 10 words of the CERAD for the diagnosis of dementia in illiterates.^2^ The CERAD test is dependent on the reading of a list of 10 words, which are read aloud by the examiner when the subject is illiterate, making this test more difficult for the illiterates who must rely only on the listening of words to memorize.^6,7^

In the hitherto performed studies, we have focused on the influence of educational level, while the influence of age and gender have not been exhaustively studied, mainly because the previous series only included elderly participants, thus precluding the analysis of the influence of age.^1-4^ In this paper we have evaluated the influence of age, gender and educational level on the performance of these tests.

## Methods

As a part of a study on functional literacy level,^8^ we evaluated a convenient sample of 325 adult volunteers using the Brief Cognitive Battery-Edu. To be included in the study the subject had to be a healthy volunteer, while the exclusion criteria were illiteracy, neurologic or psychiatric disorders, visual or hearing impairment, untreated chronic clinical conditions, alcoholism, use of drugs, and for those aged 65 or over, an informant report of cognitive or functional impairment characterized by a score higher than one in the Functional Activities Questionnaire (Pfeffer et al., 1982).^9^

There were 207 women and 118 men, having a mean age of 47.1 (±16.8) years, ranging from 19 to 81 years. Education was characterized by the years of school going and had a mean 9.8 (±5.0) schooling-years.

This study was approved by the Ethics Committee of the Hospital das Clínicas of the University of São Paulo School of Medicine. All subjects were informed about the objectives and procedures involved in the study and written informed consent was obtained prior to the interviews and tests.

Statistical analyses were performed with non-parametric methods because the data did not follow a normal distribution. The Mann-Whitney test was used to compare the scores obtained in the seven tests according to gender, and Spearman’s correlation was used to investigate the relationship between the scores in the tests with age and with education. For investigating the specific role of either age or education in each of the tests, we used logistic regression, establishing the scores as dependent variables, which were classified into two categories: below the median, and greater or equal to the median. The significance level adopted was 5%. The software SPSS, version 10.0, was used for analysis.

## Results

The data on age and education of the individuals are depicted in Table 1.

**Table 1:** Distribution of participants by age and educational level.

Age (years)	Educational level (years of schooling)				
	**1-3**	**4-7**	**8-11**	≥**12**	**Total**
19-34	4	12	28	53	97
35-49	11	15	24	32	82
50-64	14	23	26	22	85
65-81	13	19	15	14	61
Total	42	69	93	121	325

There were no differences between gender regarding age and educational level. When the scores of men and women in each test were compared there was a difference only in the learning test (the last of the three encoding steps), in which women had a better performance than men (p=0.012; Mann-Whitney test).

The correlations between the scores in the tests and age and educational level are shown in Table 2.

**Table 2:** Correlations between scores in the tests with age and with educational level (years of schooling).

Test	Age			Educational level	
	**Rho**	**p**		**Rho**	**p**
Visual identification	-0.089	0.110		0.151	0.006
Naming	-0.034	0.538		0.099	0.074
Incidental memory	-0.252	<0.001		0.141	0.011
Immediate memory	-0.393	<0.001		0.244	<0.001
Learning	-0.278	<0.001		0.256	<0.001
Delayed recall	-0.339	<0.001		0.231	<0.001
Recognition	-0.174	0.002		0.290	<0.001

There were significant negative correlations between age and the scores in most of the tests, except for visual identification and naming. On the other hand, there were significant positive correlation between schooling years and scores in most tests. However, there was a significant negative correlation between age and schooling-years (rho= -0.323; p<0.001).

Logistic regression showed that when the variables gender, age, educational level and education by age interaction were investigated, gender, age and educational level were included as significant variables. Gender only had influence on the last step of the encoding phase, age influenced incidental memory, immediate memory, learning and delayed recall of the drawings, whilst schooling-years had an influence on visual identification, immediate memory, learning, delayed recall and recognition of the drawings (Table 3).

**Table 3:** Logistic regression with dependent variables (tests) classified into two categories: less than the median, and equal to or greater than the median.

Test	Variables in the equation	B	Standard error	p	Exp. (B)	95% CI for Exp. (B)
Visual identification	Education (yos)	0.232	0.096	0.015	1.262	1.045-1.523
Naming	None	-	-	-	-	-
Incidental memory	Age	-0.030	0.008	<0.001	0.971	0.956 -0986
Immediate memory	Education (yos)	0.076	0.025	0.002	1.079	1.027-1.133
	Age	-0.040	0.008	<0.001	0.960	0.946-0.975
Learning	Education (yos)	0.078	0.024	0.001	1.082	1.031-1.135
	Age	-0.023	0.007	0.002	0.977	0.963-0.991
	Gender	0.634	0.246	0.010	1.886	1.164-3.054
Delayed recall	Education (yos)	0.075	0.026	0.004	1.077	1.024-1.133
	Age	-0.045	0.008	<0.001	0.956	0.941-0.972
Recognition	Education (yos)	0.199	0.042	<0.001	1.220	1.124-1.325

yos: years of schooling.

## Discussion

The main finding of this study was confirmation that both age and educational level influence even simple tests such as these from the BCB-Edu.

Age and educational level have an influence on different types of tests of the BCB-Edu. Age influences the performance in incidental memory, immediate memory, learning and recall of the drawings, while educational level does not influence the incidental memory, but has a wider influence on performance, from visual identification to recognition of the drawings, including the other memory tests that were also influenced by age. Both age and educational level did not influence the naming of the drawings once they were identified, probably reflecting that this constituted a very easy test.

In a study of the effect of age and educational level on the performance in the Mini-Mental State Examination (MMSE), Ishizaki et al. found that both age and educational level have an influence on the global scores, as well as on all specific items of the test, except for naming.^10^ In another study, the recall of three words of the MMSE was also considered sensitive to age.^11^

Episodic memory decreases with age, due to either deficits of encoding or retrieval, or both.^12^ Our findings are consistent with retrieval deficits because age did not influence the recognition of the drawings, although we cannot exclude that encoding may also have been decreased by age. In the encoding phase of the BCB-Edu, the performance was indeed influenced by age, but one should bear in mind that the performance in the encoding phase is analyzed through the retrieval of the names of the objects that had been seen as drawings. In this study, incidental memory was more influenced by age than by educational level, while immediate memory was influenced by both age and educational level. Incidental memory may be related to immediate memory or may be associated with working memory, which in turn has been shown to be influenced by aging.^13,14^

Educational level had influenced more tests of the BCB-Edu than age in this study. Visual identification of the drawings was influenced by educational level, while naming was not. In order to understand these findings, it should be explained that in the BCB-Edu, the participant is requested to name 10 objects that are presented as simple drawings on a sheet of paper and if naming is right, visual identification is also considered to be right. Errors in naming may be due to visual misidentification (where naming is not really wrong) or to true naming errors. For instance, the airplane drawing may be named as a fish, the turtle drawing as a tank or the comb drawing may be not identified at all in visuoperceptual errors, while the name of the comb may not be remembered but the patient may imitate its use in cases of naming error.

Visual identification is not a difficult task in this battery but was influenced by educational level. Non-educated subjects have difficulties in visual discrimination, evident in superimposed figures,^15,16^ in naming two-dimensional objects^17^ and in differences in naming black and white objects from drawings and photos.^18^ Ardila et al. conducted a cross-sectional study of Mexican adults aged between 16 and 85; they found that verbal memory performance declined more markedly with age among illiterates than among subjects with more than ten years of schooling.^19^

Our recognition sub-test has suffered an influence from both education and age, a finding replicated by other authors, with worse recognition among elders observed,^20^ while another report reported no age influence in the recognition test or cued recall, although age had great power in determining retrieval scores.^21^

Ishizaki et al.^10^ found that the effect of gender was not significant in the global scores and specific items of the MMSE. However, similarly to our findings, other studies have reported better performance of women in memory tests.^22-24^ In one study using the California Verbal Learning Test, women outperformed men in trial 5, which corresponds to the learning or last step of the acquisition or encoding phase of the test, but gender was also shown to influence immediate memory and delayed recall tests.^22^

Some limitations should be outlined: the particular influence of age and educational level was more difficult to analyze because in this sample the educational level was lower in the more aged individuals, a common finding in population studies. As both advancing age and low education may predispose to poorer performance in neuropsychological tests, their particular influence should not be difficult to disentangle. The main limitation of this study is the absence of illiterate individuals in the sample, because it is well known that illiteracy has a great influence on the performance of neuropsychological tests, where the effect of education on performance is best represented by a negatively accelerated curve.^25^ Another limitation is a lack of very old and illiterate subjects in this sample, which would have allowed a wider scope of analysis possibilities.

In summary, age and education have a significant correlation with performance in memory related tests of the BCB-Edu, although the extent of their influence differs according to the nature of the memory test.
